# Polarisation-insensitive generation of complex vector modes from a digital micromirror device

**DOI:** 10.1038/s41598-020-66799-9

**Published:** 2020-06-26

**Authors:** Carmelo Rosales-Guzmán, Xiao-Bo Hu, Adam Selyem, Pedro Moreno-Acosta, Sonja Franke-Arnold, Ruben Ramos-Garcia, Andrew Forbes

**Affiliations:** 1 Wang Da-Heng Collaborative Innovation Center for Quantum manipulation & Control, Harbin University of Science and Technology, Harbin, 150080 China; 2 Fraunhofer Centre for Applied Photonics, G1 1RD Glasgow, Scotland; 3 Instituto Nacional de Astrofísica, Óptica y Electrónica, Luis Enrique Erro 1, Tonantzintla, Puebla México; 4 School of Physics and Astronomy, University of Glasgow, G12 8QQ Glasgow, Scotland; 5 School of Physics, University of the Witwatersrand, Johannesburg, 2050 South Africa

**Keywords:** Displays, Optics and photonics, Lasers, LEDs and light sources, Optical techniques

## Abstract

In recent time there has been an increasing amount of interest in developing novel techniques for the generation of complex vector light beams. Amongst these, digital holography stands out as one of the most flexible and versatile with almost unlimited freedom in the generation of scalar and complex vector light fields featuring arbitrary polarisation distributions and spatial profiles. In this manuscript we put forward a novel technique, which relies on the polarisation-insensitive attribute of Digital Micromirror Devices (DMDs). In a prior work where we outlined a new detection scheme based on Stokes projections we alluded to this technique. Here we outline the creation process in full, providing all the details for its experimental implementation. In addition, we fully characterise the performance of such technique, providing a quantitative analysis of the generated modes. To this end, we experimentally reconstruct the transverse polarisation distribution of arbitrary vector modes and compare the ellipticity and flatness of the polarisation ellipses with theoretical predictions. Further, we also generate vector modes with arbitrary degrees of non-separability and determine their degree of concurrence comparing this to theoretical predictions.

## Introduction

Complex vector light fields are fascinating states of light that have captured the interest of researchers across a wide variety of fields where they have found a myriad of applications^1,2^. In vector light fields the spatial and polarisation degrees of freedom are coupled in a non-separable way, as in tightly focused beams but not to be confused with these, giving rise to a non-homogeneous transverse polarisation distribution that holds many interesting properties^3–7^. This non-separability has been identified as the classical analogue of local quantum entanglement, enabling quantum-like phenomena at the classical level^8–14^. In the last decade several techniques have been proposed to generate vector beams, including interferometric arrays^15–18^, liquid crystal wave plates^19,20^, glass cones^21,22^, metamaterials^23^, Spatial Light Modulators (SLMs)^24–31^ and more recently Digital Micromirror Devices (DMDs)^32–35^. Ultimately, most techniques aim for the full control over the phase, amplitude and polarisation of light towards the generation of arbitrary vector modes, being computer-controlled devices one of the most flexible and versatile technologies. Crucially, while SLMs are polarisation dependent, allowing only the modulation of linear polarisation (typically horizontal), DMDs can modulate any polarisation, a property that has gone almost unnoticed since common experimental setups still resemble those associated to polarisation-dependent SLMs. That is, in order to generate arbitrary vector beams with an SLM, the transverse profiles of both polarisation components have to be manipulated independently, either in interferometric arrays containing one or two SLMs^16,18,25,29,31,36^, or via a temporal sequence using a double pass over a single SLM^30,37^. Crucially, the DMD technology allows the generation of complex light fields at high refresh rates (~30 KHz) and over a broad band of the visible spectrum of monochromatic sources^38^.

In this manuscript, we put forward a compact and robust technique for the generation of arbitrary vector modes, which takes full advantage of the properties of DMDs, in particular their polarisation-insensitive attribute. In our prior work^39^ we outlined a new detection scheme based on Stokes projections, only alluding to the ability to create such beams from DMDs. Here we outline the creation process in full, highlighting its performance through a quantitative characterisation of the generated modes. Our device comprises the illumination of a DMD with two beams of orthogonal polarisation impinging at different angles to modulate the spatial degree of freedom of both polarisation components in a single pass and independent to each other. For this purpose, we display on the DMD a binary multiplexed hologram formed by the superposition of two independent holograms overlapped with unique linear phase gratings designed to ensure the overlap of the first diffraction order along a common propagation axis, where the vector mode is generated. Given that both constituent holograms are independent, our all-digital technique can generate high-quality vector modes with tunable degrees of non-separability, and arbitrary spatial and polarisation distributions. We demonstrate this by performing an exhaustive evaluation of the generated modes. First, we reconstruct experimentally the transverse polarisation distribution of arbitrary vector modes and compare the flattening and rotation angle of each polarisation ellipsis on a 18 × 18 grid with theoretical predictions^40^. Second, we generate vector modes with increasing degrees of non-separability, which we measure experimentally through the degree of concurrence^39,41,42^, to estimate the degree of accuracy of our device.

## Generation of cylindrical vector vortex modes

It is well-known that the spatial degree of freedom of vector modes can be encoded using any set of solutions of the wave equation in its exact or paraxial approximation. Common examples of such are, Bessel- Laguerre-, Mathieu-, Ince or Airy-Gaussian vector modes^32,37,43–47^. Amongst these, vector beams with cylindrical symmetry, commonly know as cylindrical vector vortex modes, have raised an increasing amount of interest, in part due to their wide variety of applications^1,43^. As such, to demonstrate our technique and without loss of generality, we will restrict our analysis to the Laguerre-Gaussian ($L{G}_{p}^{\ell }$) modes, natural solutions of the paraxial wave equation of the cylindrical coordinates ($\rho$, $\varphi$)^48^. Such modes are characterised by an azimuthally-varying phase of the form $\exp (i\ell \varphi )$, where the index $\ell \in {\mathbb{Z}}$, known as the topological charge, is associated to the number of times the phase wraps around the optical axis where for $\ell \ne 0$ it becomes singular. Such singularity gives rise to an intensity null along the optical axis, producing ring-shaped light beams commonly-known as optical vortices. Further, such beams carry a well-defined amount of orbital angular momentum $\ell \hslash$ per photon, where $\hslash$ is the reduced Plank’s constant^49,50^. The index $p\in {\mathbb{N}}$ is responsible for the generation of ($p+1$) intensity rings along the radial direction. As per the polarisation DoF, in principle we can use any orthogonal bsis, namely, linear, diagonal, circular or elliptical. Here and without the loss of generality, we will use the circular polarisation basis defined as, $\hat{r}=(\hat{h}+i\hat{v})/\sqrt{2}$ and $\hat{l}=(\hat{h}-i\hat{v})/\sqrt{2}$, where $\hat{h}$ and $\hat{v}$ represent the unitary vectors of the horizontal and vertical polarisation basis, respectively. Hence, using the above described spatial an polarisation DoF, cylindrical vector vortex modes can be described mathematically as^36,51^,

1 $$\overrightarrow{u}(\rho ,\varphi )=\,\cos \,(\theta )\,L{G}_{{p}_{1}}^{{\ell }_{1}}(\rho ,\varphi ){{\rm{e}}}^{i\alpha }\hat{r}+\,\sin \,(\theta )\,L{G}_{{p}_{2}}^{{\ell }_{2}}(\rho ,\varphi ){{\rm{e}}}^{-i\alpha }\hat{l},$$

where, the coefficients $\cos (\theta )$ and $\sin (\theta )$ ($\theta \in [0,\pi /2]$) are weighting factors that allow a smooth transition of the field $\overrightarrow{u}(\rho ,\varphi )$, from scalar ($\theta =0$ and $\theta =\pi /2$) to vector ($\theta =\pi /4$)^39^. In addition, the term ${{\rm{e}}}^{i\alpha }$ ($\alpha \in [\,-\,\pi /2,\pi /2]$) generates a phase difference between both polarisation components.

**Figure 1:**
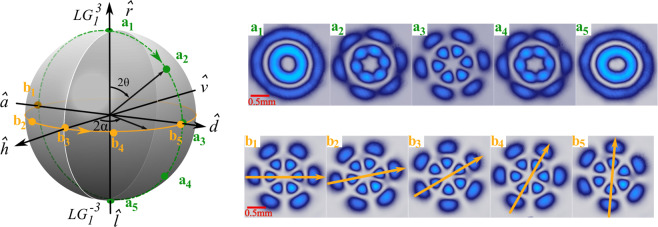
Representative examples of vector modes shown as points along the green-dashed and solid-yellow lines drawn on the HOPS, labelled as (a_1_–a_5_ and b_1_–b_5_), respectively. Their corresponting experimental intensity profiles, acquired by passing these through a linear polariser, are shown on the left panel, see also Visualisation 1 and Visualisation 2. Here, $\hat{h}$, $\hat{v}$, $\hat{d}$, $\hat{a}$, $\hat{r}$ and $\hat{l}$ represent the horizontal, vertical, diagonal, antidiagonal, right- and left-handed unitary polarisation vectors.

To begin with, we will show that our technique enables the generation of arbitrary vector modes on the Higher-Order Poincaré Sphere (HOPS). To this end, lets recall that vector modes given by Eq. 1 are mapped to unique positions ($2\alpha$, $2\theta$) on the surface of a unitary sphere in which, the north and south poles are assigned to the scalar modes $L{G}_{{p}_{1}}^{{\ell }_{1}}(\rho ,\varphi )\hat{r}$ and $L{G}_{{p}_{2}}^{{\ell }_{2}}(\rho ,\varphi )\hat{l}$, respectively^52^. Further, points along the equator correspond to pure vector beams while the remaining to vector modes with elliptical polarisation. Figure 1 shows representative examples of vector modes generated with our device, represented on the HOPS. For these examples we used $L{G}_{1}^{3}(\rho ,\varphi )\hat{r}$ and $L{G}_{1}^{-3}(\rho ,\varphi )\hat{l}$. The top-right insets of Fig. 1(a_1_–a_5_) show the intensity profile of modes generated along the dashed line (green) that connects the North and South poles, which were generated according to Eq. 1 by keeping *α* constant while varying $\theta \in [0,\pi /2]$ (see also Visualisation 1). The bottom-right insets (b_1_–b_5_) show representative examples of the modes generated along the equatorial solid line (yellow) generated by keeping *θ* constant while changing $\alpha \in [\,-\,\pi /4,\pi /4]$ (See also Visualisation 2). The intensity profiles were obtained by passing the vector beams through a linear polariser.

## Characterisation of vector beams

### Characterisation through Stokes polarimetry

**Figure 2:**
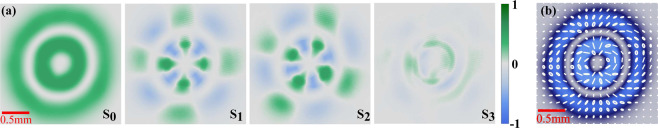
Experimental reconstruction of polarisation using Stokes polarimetry. (**a**) Example of the stokes parameters S_0_, S_1_, S_2_ and S_3_ used to reconstruct the polarisation distribution of the vector mode $\frac{1}{\sqrt{2}}(L{G}_{1}^{-2}\hat{r}+L{G}_{1}^{2}\hat{l})$ shown in (**b**), where the local polarisation is indicated on an 18 × 18 grid using polarisation ellipses.

In order to quantify the capabilities of our generation technique, we programmed on the DMD various Poincaré beams obtained from different combinations of ${p}_{1}$, ${\ell }_{1}$, ${p}_{2}$, and ${\ell }_{2}$^36^. Afterwards we reconstructed their transverse polarisation distribution, through Stokes polarimetry, and compared with theory. The Stokes parameters were computed from a set of four intensity measurements as^53^,

2 $${S}_{0}={I}_{0},\,{S}_{1}=2{I}_{h}-{S}_{0},\,{S}_{2}=2{I}_{d}-{S}_{0},\,{\rm{and}}\,{S}_{3}=2{I}_{r}-{S}_{0},$$

where ${I}_{0}$ is the total intensity along the transverse plane, and ${I}_{h}$, ${I}_{d}$ and ${I}_{r}$ the intensity of the horizontal, diagonal and right-handed polarisation components, respectively. Experimentally, ${I}_{h}$ and ${I}_{d}$ can be measured by passing the generated vector mode $\overrightarrow{u}(\rho ,\varphi )$ through a linear polariser at Θ = 0° and Θ = 45°, respectively, while the intensity of the ${I}_{r}$ polarisation component by passing it simultaneously through a QWP at $\beta =45^\circ$ and a linear polariser at Θ = 90°. Figure 2(a) shows an example of the Stokes parameters obtained for the specific case $(L{G}_{1}^{-2}\hat{r}+L{G}_{1}^{2}\hat{l})/\sqrt{2}$, where $\alpha =0$. The reconstructed polarisation is shown in Fig. 2(b), featuring a spider-like distribution.

**Figure 3:**
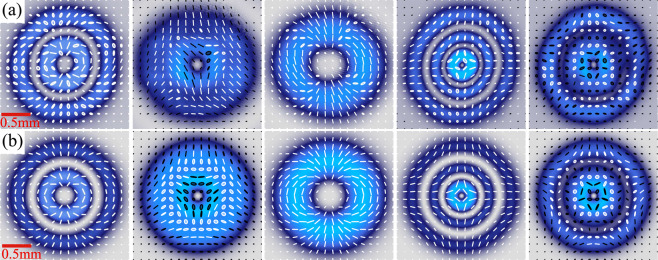
Experimental (**a**) and theoretical (**b**) reconstruction of the transverse polarisation distribution of a set of vector modes given by pairs of scalar $L{G}_{p}^{\ell }$ modes, from left to right, $\{L{G}_{1}^{-2},L{G}_{1}^{+2}\}$, $\{L{G}_{0}^{+2},L{G}_{1}^{+1}\}$, $\{L{G}_{0}^{-2},L{G}_{0}^{+2}\}$, $\{L{G}_{2}^{+1},L{G}_{2}^{-1}\}$ and $\{L{G}_{1}^{+2},L{G}_{2}^{-1}\}$.

In Fig. 3(a) we show experimental examples of the transverse polarisation distribution, reconstructed as explained before, of a representative set of vector modes defined by the pairs of scalar $L{G}_{p}^{\ell }$ modes, from right to left, $\{L{G}_{1}^{-2},L{G}_{1}^{+2}\}$, $\{L{G}_{0}^{+2},L{G}_{1}^{+1}\}$, $\{L{G}_{0}^{-2},L{G}_{0}^{+2}\}$, $\{L{G}_{2}^{+1},L{G}_{2}^{-1}\}$ and $\{L{G}_{1}^{+2},L{G}_{2}^{-1}\}$. These modes are compared to their theoretical counterpart shown in Fig. 3(b). Here, white and black ellipses represent right and left circular polarisation, respectively. A straight comparison of the experimentally reconstructed polarisation ellipses with theoretical predictions provides information about the quality of our generated modes. This can be done by comparing the flattening (*f*) and orientation (*a*) of the experimental and theoretical polarisation ellipse across the entire transverse plane. To this end, we computed *f* and *α* on an 18 × 18 grid as^40^,

3 $$f=\frac{\sqrt{{S}_{0}+\sqrt{{S}_{1}^{2}+{S}_{2}^{2}}}-\sqrt{{S}_{0}-\sqrt{{S}_{1}^{2}+{S}_{2}^{2}}}}{\sqrt{{S}_{0}+\sqrt{{S}_{1}^{2}+{S}_{2}^{2}}}}\,{\rm{and}}\,\alpha =\frac{1}{2}\,\arctan \,\left(\frac{{S}_{2}}{{S}_{1}}\right),$$

respectively, and computed the Root Mean Squared Error (RMSE) between experiment and theory. The flattening is a number between 0 and 1 that measures the eccentricity (shape) of the polarisation ellipse, 0 for circular polarisation and 1 for linear, while the parameter $\alpha$ measures its orientation. Table 1 shows the averaged RMSE across the transverse plane, for each of the vector modes shown in Fig. 3. Notably, both the RMSE_*α*_ and the RMSE_*f*_ are relatively small, not exceeding 7% and 2%, respectively. It is worth mentioning that the quality of the modes presented here can be further enhanced in two ways. Firstly, their elliptical shape, caused by phase distortions arising from aberrations produced by the DMD’s screen, which in general is not optically flat, can be measured and compensated for using an interferometric technique as detailed in^34^. Secondly, the polarisation distribution can be corrected by finely adjusting the coaxial superposition of both beams, which can be done digitally by tuning the frequency of the linear diffraction grating of each beam.

**Table 1:** RMSE_*α*_ and RMSE_*f*_ of the Poincaré modes shown on Fig. 3.

Vector mode	$\{L{G}_{1}^{-2},L{G}_{1}^{+2}\}$	$\{L{G}_{0}^{+2},L{G}_{1}^{+1}\}$	$\{L{G}_{0}^{-2},L{G}_{0}^{+2}\}$	$\{L{G}_{2}^{+1},L{G}_{2}^{-1}\}$	$\{L{G}_{1}^{+2},L{G}_{2}^{-1}\}$
RMSE_*α*_	5.52%	6.31%	5.33%	5.50%	5.68%
RMSE_*f*_	1.39%	1.53%	1.26%	1.32%	1.36%

### Characterisation through concurrence

As stated earlier, our device can generate any complex mode on the HOPS, from vector to scalar, as such, in this section we provide specific numbers to show its accuracy in generating vector modes with tunable degrees of non-separability. To this end, we will use a well-known technique that exploits the similarities between classical and quantum local entanglement, concurrence (*C*). Concurrence has been identified as a proper tool to measure the degree of non-separability of vector beams, which has been termed Vector Quality Factor (VQF). The VQF assigns values in the interval $[0,1]$ to the degree of coupling between the spatial and polarisation degrees of freedom, 0 for scalar and 1 for vector modes^41,42^. This technique comprises the projection of the vector mode onto one degree of freedom, polarisation in our case, which is later passed through a series of phase filters that performs a projection onto the spatial degree of freedom. Explicitly, the VQF is determined as^41^

4 $${\rm{VQF}}={\rm{Re}}\{C\}={\rm{Re}}\{{(1-\sum _{i}{\langle {\sigma }_{i}\rangle }^{2})}^{1/2}\},$$

where $\langle {\sigma }_{1}\rangle$, $\langle {\sigma }_{2}\rangle$ and $\langle {\sigma }_{3}\rangle$ are the expectation values of the Pauli operators. To measure this value experimentally, the two circular polarisation components are first split into their left and right polarisation components, which propagate along different trajectories, this can be done, for example, with a polarisation grating. The resulting beams are then projected onto a set of six phase holograms that performs the projection onto the spatial degree of freedom. The holograms, encoded on an SLM, consist of helical phases, two with topological charges $\ell$ and $-\,\ell$ plus four superposition of the same, namely, $\exp \,({\rm{i}}\ell \varphi )+\exp \,({\rm{i}}\gamma )\,\exp \,(\,-\,{\rm{i}}\ell \varphi )$ with $\gamma =\{0,\pi /2,\pi ,3\pi /2\}$. The 12 intensities ${I}_{ij}$ are then measured as the on-axis values of the far-field intensity recorded on a Charge-Coupled Device (CCD) camera. For the sake of clarity, the 12 required intensity measurements are explicitly shown in Table 2. Incidentally, the number of required measurements can be reduced to a minimum of 8 by projecting first over the spatial degree of freedom followed by a projection onto the polarisation degree of freedom^54^.

**Table 2:** Normalised intensity measurements *I*_*ij*_ to determine the expectation values 〈*σ*_*i*_〉.

Basis states	${\ell }^{+}$	${\ell }^{-}$	*γ*_0_	*γ*_*π*/2_	*γ*_*π*_	*γ*_3*π*/2_
*r*	${I}_{r{\ell }^{+}}$	${I}_{r{\ell }^{-}}$	${I}_{r{\gamma }_{0}}$	${I}_{r{\gamma }_{\pi /2}}$	${I}_{r{\gamma }_{\pi }}$	${I}_{r{\gamma }_{3\pi /2}}$
*l*	${I}_{l{\ell }^{+}}$	${I}_{l{\ell }^{-}}$	${I}_{l{\gamma }_{0}}$	${I}_{l{\gamma }_{\pi /2}}$	${I}_{l{\gamma }_{\pi }}$	${I}_{l{\gamma }_{3\pi /2}}$

Here, for example, ${I}_{r{\ell }^{+}}$ represents the intensity of the right circular polarisation component after its projection on the $\exp \,({\rm{i}}\ell \phi )$ phase filter. The expectation values ${\sigma }_{i}$ are explicitly computed from the twelve intensity measurement ${I}_{ij}$ as,

5 $$\begin{array}{rcl}\langle {\sigma }_{1}\rangle  & = & ({I}_{r{\gamma }_{0}}+{I}_{l{\gamma }_{0}})-({I}_{r{\gamma }_{\pi }}+{I}_{l{\gamma }_{\pi }}),\\ \langle {\sigma }_{2}\rangle  & = & ({I}_{r{\gamma }_{\pi /2}}+{I}_{l{\gamma }_{\pi /2}})-({I}_{r{\gamma }_{3\pi /2}}+{I}_{l{\gamma }_{3\pi /2}}),\\ \langle {\sigma }_{3}\rangle  & = & ({I}_{r{\ell }^{+}}+{I}_{l{\ell }^{+}})-({I}_{r{\ell }^{-}}+{I}_{l{\ell }^{-}}).\end{array}$$

**Figure 4:**
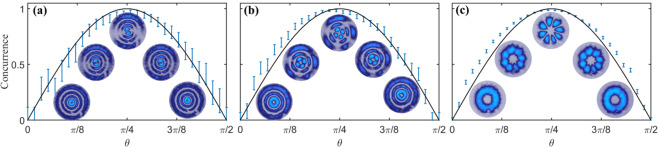
Experimental verification of the concurrence *C* as function of the modal weighting *θ* that allows the transition between scalar and vector modes. The cases shown here correspond to vector modes generated by the combinations (**a**) $\{L{G}_{3}^{-1},L{G}_{3}^{1}\}$, (**b**) $\{L{G}_{2}^{-2},L{G}_{2}^{2}\}$ and (**c**) $\{L{G}_{0}^{-3},L{G}_{0}^{3}\}$, for $\alpha =0$. The insets show the intensity profile after a linear polariser for $\theta =[0,\pi /8,\pi /4,3\pi /8,\pi /2]$.

Figure 4 shows representative examples of the concurrence measured for three different modes, as function of the weighting factor $\theta \in [0,\pi /2]$, which allows a monotonic variation from scalar to vector. The specific modes are, $(L{G}_{3}^{-1}\hat{r}+L{G}_{3}^{1}\hat{l})/\sqrt{2}$, $(L{G}_{2}^{-2}\hat{r}+L{G}_{2}^{2}\hat{l})/\sqrt{2}$ and $(L{G}_{0}^{-3}\hat{r}+L{G}_{0}^{3}\hat{l})/\sqrt{2}$, Fig. 4(a–c), respectively. To obtain the transition from scalar to vector, we varied digitally the amplitude coefficients determined by $\theta \in [0,\pi /2]$. The insets of each plot show the recorded intensity distribution of the input field $u(\overrightarrow{r})$ at the specific values $\theta =[0,\pi /8,\pi /4,3\pi /8,\pi /2]$, after passing through a linear polariser. Notably, Small intensity fluctuations at the detector caused uncertainty in the measured concurrence (shown as error bars in Fig. 4) which was characterised for each intensity measurement by taking the standard deviation after averaging over the 64 central pixels. As an additional comment, the error bars increase as the radial index *p* of the $L{G}_{p}^{\ell }$ modes increase, this can be attributed to the fact that the spatial projection was performed on the azimuthal index $\varphi$ ignoring the radial index. One way to solve this, is by using our recently proposed basis-independent technique which allows to measure the concurrence *C* directly from the Stokes parameters^39^. Specifically, *C* can be measured as,

6 $$C=\sqrt{1-{\left(\frac{{{\mathbb{S}}}_{1}}{{{\mathbb{S}}}_{0}}\right)}^{2}-{\left(\frac{{{\mathbb{S}}}_{2}}{{{\mathbb{S}}}_{0}}\right)}^{2}-{\left(\frac{{{\mathbb{S}}}_{3}}{{{\mathbb{S}}}_{0}}\right)}^{2}},$$

where ${{\mathbb{S}}}_{i}$ are the values of the Stokes parameters ${S}_{i}$ integrated over the entire transverse profile. A complete explanation of this technique as well as additional experimental measurements are given in^39^.

## Discussion

The approach outlined in this manuscript, which is capable to generate arbitrary vector modes using a single DMD, takes full advantage of their polarisation-insensitive attribute that allows them to modulate any polarisation state. Even though DMDs have been around for several decades it is only in recent time that they became an alternative device for the generation of scalar beams. Importantly, their use in the generation of vector beams is quite recent and therefore, most approaches still resemble the experimental setups which make use of polarisation-sensitive spatial light modulators. That is, the polarisation-insensitive property of DMDs has not been properly exploited, and this is precisely the key factor in our approach. To this end, we illuminate the DMD with two beams of orthogonal polarisation impinging at two different angles, this is achieved by splitting a diagonally polarised beam, either with a Wollaston prism or a polarising beam splitter, into their two orthogonal polarisation components. Further, the DMD is addressed with a binary amplitude multiplexed hologram consisting of two independent holograms, each superimposed with a linear phase gradient that diffracts the first diffraction order along a specific angle. Each hologram encodes the amplitude and phase of a desired target mode, in our case the $L{G}_{p}^{\ell }$ modes, which can be controlled independently. The frequency of each linear phase grating is carefully selected to ensure the spatial overlap of the first diffraction order of each beam along a common propagation axis, where the complex vector mode is generated.

Notably, our device is very compact and of easy implementation, all at an affordable cost, at least one order of magnitude cheaper than liquid crystal spatial light modulators. Nonetheless, it is very powerful, enabling the generation of arbitrary complex light modes with unlimited spatial and polarisation distributions. Here, as a proof-of-principle we generated $L{G}_{p}^{\ell }$ cylindrical vector modes but, as it has been demonstrated, DMDs can generate scalar modes with almost unlimited freedom^32,55–57^. Therefore DMDs can generate vector modes with arbitrary transverse spatial profiles. Here we further demonstrated that the quality of the vector modes generated with this specific device is very high. We show this by comparing the flatness and orientation of the reconstructed polarisation ellipses across the entire transverse plane with theoretical predictions. We did this through the RMSE, finding error values lower than 7% for the flatness and lower than 2% for the orientation of the polarisation ellipses. Importantly, the ellipticity in the spatial shape of the generated modes can be corrected by compensating for the optical aberrations introduced by the screen of the DMD. In addition their polarisation distribution can also be improved by adjusting digitally the overlap between both constituting beams. We also generated cylindrical vector modes, as defined on the higher-order Poincaré sphere with arbitrary degrees of non-separability and quantified this through the concurrence *C*. We measured this using a well-known method that relies on projecting the vector mode onto the polarisation degree of freedom, followed by its projection onto a series of phase filters that perform a projection on the spatial degree of freedom. Our measurements indicate our device can generate vector beams with a choice of VQF within ±5% of the theoretical VQF when beams are close to fully vectorial, and presumably performs just as well at low VQF, but our measurement technique has low signal-to-noise ratio there. In addition, the projection of the vector modes into the spatial degree of freedom was performed considering only the azimuthal degree of freedom, ignoring the radial one. This could explain why the concurrence plots shown in Fig. 4 feature higher error bars for the cases of higher radial indices (*p*) of the $L{G}_{p}^{\ell }$. This is a problem related to our measuring technique rather than to the quality of the generation method, this measuring problem could be solved by using for example a basis-independent measure of concurrence^39^. Finally, it is worth mentioning that the modulation efficiency of DMDs is quite low, compared to spatial light modulators, and in general, the first diffraction order contains around 10% of the input power. In our case, we achieved values smaller than 5$\%$, which can be attributed to factors such as, absorption of the different optical components. Nonetheless, in the cases where efficiency can be traded out for higher refresh rates, DMDs are a great alternative.

## Methods

**Figure 5:**
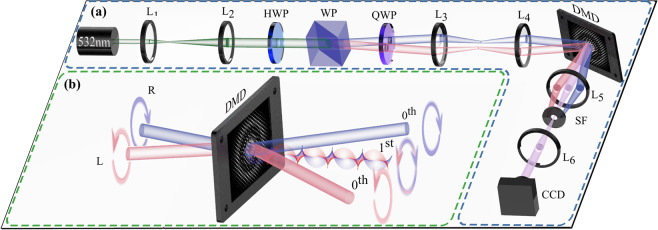
Schematic representation of our polarisation-insensitive experimental setup. (**a**) A 500 mW horizontally polarised beam ($\lambda =532\,nm$), collimated and expanded through lenses ${L}_{1}$ and ${L}_{2}$, is converted to a diagonal polarisation state by use of a half wave-plate (HWP). Afterwards, a Wollaston prism (WP) separates it into its vertical and horizontal polarisation components. A quarter wave-plate (QWP) then changes these to the circular polarisation basis. A telescope formed by lenses ${L}_{3}$ and ${L}_{4}$ redirects both beams to a digital micromirror device (DMD), impinging at different angles but onto the same geometric point of a digital hologram displayed on the DMD, as shown in (**b**). The hologram is the result of multiplexing two independent holograms with unique spatial carrier frequencies, carefully selected to ensure the overlap of the first diffracted order from each beam along a common propagation axis. Higher diffraction orders are removed using a telescope, formed by lenses ${L}_{5}$ and ${L}_{6}$, in combination with a Spatial Filter (SF). The intensity profile was recorded with a CCD (1.55 *μ*m pixel size).

Our proposal to generate arbitrary vector light fields relies on the fact that DMDs can modulate any polarisation state and therefore can tailor simultaneously, polarisation, phase and amplitude. To better understand our approach, Fig. 5(a) shows a schematic representation of our device. Here, a horizontally polarised laser beam ($\lambda =523$ nm, 500 mW) is expanded and collimated by lenses L_1_ and L_2_ (with focal length ${f}_{1}=20$ mm and ${f}_{2}=200$ mm). Afterwards, a half-wave plate at 45° rotates its polarisation to the diagonal state ${\overrightarrow{u}}_{0}(x,y)={u}_{0}(x,y)(\hat{h}+\hat{v})/\sqrt{2}$. A Wollaston prism (WP) separates the beam according to their polarisation components into two beams, separated approximately by 1.5°, one with horizontal polarisation and the other with vertical. A quarter-wave plate (QWP) placed immediately after the Wollaston prism changes the horizontal and vertical polarisation to the circular polarisation basis $\hat{l},\hat{r}$. A 4*f* imaging system composed by lenses L_3_ and L_4_ (of focal length ${f}_{3}={f}_{4}=200$ mm) redirects both beams towards the centre of a DMD (DLP Light Crafter 6500 from Texas Instruments), where they impinge under slightly different angles but are spatially overlapped. The DMD displays a multiplexed binary amplitude hologram, consisting of the superposition of two independent holograms corresponding to the desired spatial wave functions of each polarisation component. Each hologram is superimposed with a linear diffraction grating, which in combination with the different input angles, ensure the overlapping of the first diffraction order along a common propagation axis, where the desired complex vector field $\overrightarrow{u}(\rho ,\varphi )$ is generated. This is illustrated in Fig. 5(b), where, for the sake of clarity, the DMD is represented as a transmission device. Both input beams with orthogonal circular polarisation impinge on the centre of the hologram displayed on DMD. At this step, the positioning of the DMD is crucial to ensure the overlap of both beams. After the DMD, the 0th diffraction order of each beam propagate diverging from each other. Nonetheless, the diffraction grating ensures the overlap of the first diffraction order of each beam along a common propagation axis, where the vector mode is generated. A spatial filter (SF) placed in the far field plane of a telescope imaging the DMD plane, realised with lenses L_5_ and L_6_ of focal lengths ${f}_{5}={f}_{6}=100$ mm, removes all higher diffraction orders leaving only the first order from each beam. For the sake of clarity, higher diffraction orders are not shown neither in Fig. 5(a), nor in Fig. 5(b). Notably, our device can generate arbitrary vector fields at high speed rates and without the mechanical movement of optical components by simply refreshing the digital holograms displayed on the DMD.

**Figure 6:**
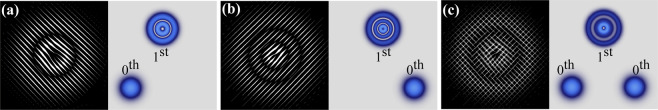
Schematic representation of the multiplexing principle. Binary hologram to generate the modes (**a**) $L{G}_{1}^{2}$ and (**b**) $L{G}_{2}^{-1}$, (left panel). The frequency value of each linear phase is properly chosen to shift the beam to the desired position, as shown on the right panel of (**a**,**b**). An example of a multiplexed hologram composed of the holograms shown on (**a**,**b**) is shown on the left panel of (**c**), whereas the overlapping of both first diffraction orders is shown on the right panel. Here, for the sake of clarity we only show the first and zero diffraction order, higher diffraction orders were omitted.

The hologram displayed in the DMD consist of the multiplexing of two binary amplitude holograms. The transmittance function ${T}_{i}(x,y)$ of each hologram is computed as^33^,

7 $$\begin{array}{c}{T}_{i}(x,y)=\frac{1}{2}+\frac{1}{2}{\rm{s}}{\rm{g}}{\rm{n}}\left\{\cos ,(,{\phi }_{i},(,x,,,y,),+,2,\pi ,(,{\nu }_{i},x,+,{\eta }_{i},y,),),+,\,,\cos ,(\arcsin \left(\frac{{A}_{i}(x,y)}{{A}_{{i}_{max}}}\right))\right\}\end{array}$$

where, sgn{u} is the sign function. The phase and amplitude of the encoded beams are denoted by ${\phi }_{i}(x,y)$ and ${A}_{i}(x,y)$, respectively, where *A*_*imax*_ is the maximum amplitude value. Further, the term $2\pi ({\nu }_{i}x+{\eta }_{i}y)$ is an additional linear phase with spatial frequency ${\nu }_{i}$ and ${\eta }_{i}$, that specifies the angle of diffraction of the first diffracted order. The position of the first diffraction order ($U,V$) in the Fourier plane, is related to the grating frequency ($\nu ,\eta$), the wavelength of the laser (*λ*) and the focal length (*f*) of the Fourier lens as, $U=\nu \lambda f$ and $V=\eta \lambda f$^28,58^. In order to generate a multiplexed hologram, we superimpose two individual holograms whose transmittance function is given by,

8 $$\begin{array}{ccc}T(x,y) & = & C\left[\frac{1}{2},+,\frac{1}{2},{\rm{s}}{\rm{g}}{\rm{n}},\{\cos ({\phi }_{1}(x,y)+{\alpha }_{1}+2\pi ({\nu }_{1}x+{\eta }_{1}y))+\cos \left(\arcsin ,(\frac{{A}_{1}(x,y)}{{A}_{{1}_{max}}})\right)\}\right]\\  &  & +S\left[\frac{1}{2},+,\frac{1}{2},{\rm{s}}{\rm{g}}{\rm{n}},\{\cos ({\phi }_{2}(x,y)+{\alpha }_{2}+2\pi ({\nu }_{2}x+{\eta }_{2}y))+\cos \left(\arcsin ,(\frac{{A}_{2}(x,y)}{{A}_{{2}_{max}}})\right)\}\right],\,\end{array}$$

were the amplitude terms $C=\cos (\theta )$ and $S=\sin (\theta )$ determines the amount of light diffracted into the first diffraction order of each mode. This is what allows our device to generate scalar beams ($\theta =0$ and $\theta =\pi /2$), vector beams ($\theta =\pi /4$) or intermediate states. The constant phases *α*_1_ and *α*_2_ allows us to change the intramodal phase between both modes. In our case to ensure an overlap of the first diffraction order of each beam, that is, $({U}_{1},{V}_{1})=({U}_{2},{V}_{2})$, we selected ${\nu }_{1}={\nu }_{2}=\nu$ and ${\eta }_{1}=-\,{\eta }_{2}=\eta$. In this particular case and taking into account the initial separation of the beams (introduced by the Wollaston prism) as well as the focal length of the Fourier lens (*f* = 100 mm), we used frequency values $\nu \approx 24\,{{\rm{mm}}}^{-1}$ and $\eta \approx 24\,{{\rm{mm}}}^{-1}$. To better clarify this, Fig. 6 shows a schematic representation of our previous description, were we also show an example of the binary holograms generated through Eq. 8. In Fig. 6(a) we show, on the left, the binary hologram that generates the mode $L{G}_{1}^{2}(\rho ,\varphi )$ whereas on the right we illustrate how a positive linear grating shifts the 1st diffraction order towards the right of the 0th diffraction order. In Fig. 6(b) we show the binary hologram required to generate the mode $L{G}_{2}^{-1}(\rho ,\varphi )$ (left panel), in this case we used a negative frequency to shift the 1st order to the left of the 0th order (right panel). Finally, in Fig. 6 we showed a multiplexed holograms composed of the previous two holograms (left panel), as result, the 1st diffraction order of each beam overlaps wich each other. The binary holograms shown here, does not correspond to the ones used in our experiment, we displayed these only to exemplify the effect of the linear phase grating. In addition and for the sake of clarity, in this schematic representation we only show the first diffraction order of each beam as well as the zero order for reference, higher diffraction orders, which inevitably appear, were omitted. Experimentally, all higher diffraction orders are removed, using a spatial filter, leaving only the first order from each constituting beam, as explained in our Methods section.

## Supplementary information

Supplementary Information 1.

Supplementary Information 2.

## Data Availability

All data regarding the work presented here is available upon reasonable request to the corresponding author.
